# High Post-Treatment Leptin Concentration as a Prognostic Biomarker of the High Risk of Luminal Breast Cancer Relapse: A Six-Year Comprehensive Study

**DOI:** 10.3390/life12122063

**Published:** 2022-12-08

**Authors:** Katarzyna Kwiatkowska, Piotr Rhone, Katarzyna Wrzeszcz, Barbara Ruszkowska-Ciastek

**Affiliations:** 1 Department of Pathophysiology, Faculty of Pharmacy, Nicolaus Copernicus University, Collegium Medicum, 85-094 Bydgoszcz, Poland; 2 Clinical Ward of Breast Cancer and Reconstructive Surgery, Oncology Centre Prof. F. Łukaszczyk Memorial Hospital, 85-796 Bydgoszcz, Poland

**Keywords:** leptin, breast cancer, relapse, adiponectin, treatment

## Abstract

(1) Background: Nowadays, obesity is well-recognised as a significant risk factor for many chronic diseases, for example, hypertension, diabetes, atherosclerosis and cancer. This study is designed to investigate the prognostic value of the pre- and post-treatment serum levels of adiponectin and leptin in luminal A and B invasive breast cancer (IBrC) patients based on six-years follow-up. (2) Methods: Among 70 patients who underwent breast surgery, 35 were Stage I and 35 were Stage II. The concentrations of pre- and post-treatment adiponectin and leptin were evaluated with a specific ELISA kit. The median follow-up was 68.5 months (inter-quartile range (IQR) = 59–72 months) with a recurrence rate of 15.71%. (3) Results: Generally, concentrations of leptin and adiponectin increased after adjuvant therapy. Follow-up showed a significantly higher incidence of disease relapse in IBrC patients with a high post-treatment concentration of leptin (25.71% vs. 5.71% of cases with a low post-treatment concentration of leptin). A post-treatment leptin concentration of 26.88 ng/mL with a specificity of 64.9% and a sensitivity of 88.9% was determined as the best cut-off value to distinguish patients with disease recurrence from those without disease relapse. (4) Conclusions: Our results demonstrated that only the post-treatment serum leptin concentration may be of value as a prognostic indicator and could contribute to predicting a future outcome for patients with early-stage IBrC.

## 1. Introduction

According to the International Agency for Research on Cancer (IARC), in 2020 invasive breast cancer (IBrC) had become the most commonly diagnosed type of cancer and the most leading cause of death in women worldwide. The IARC estimates that the incidence of breast cancer will increase by more than a third, to over three million new cases annually by 2040. This increase will be mainly due to demographic factors and lifestyle changes [1]. It is estimated that approximately one-third of postmenopausal breast cancers are associated with a potentially modifiable factor, that is, the accumulation of adipose tissue which is metabolically overactive [2]. In 2000, there were dynamic developments in the technology of genomic analysis and identification of gene expression, which resulted in the approval of a molecular classification of breast cancers. There are now four main subtypes of breast cancer: luminal A, luminal B, non-luminal human epidermal growth factor receptor 2 (HER2) positive and triple-negative. The classification is based on the expression of breast cancer markers, i.e., oestrogen receptor (ER), progesterone receptor (PR), HER2 and the proliferation marker Ki-67 expressions [3]. The value of Ki-67, which distinguishes luminal A from luminal B tumours, can vary. At a conference in Saint Gallen in 2011, the cut-off point for Ki-67 was set at 14% based on the median; however, in 2013 it was found that a cut-off point only of 20% indicates a higher proliferation risk [4].

Interestingly, adipose tissue is the main component of the breast, but the percentage of adipose tissue varies from person to person, depending on the variability of the lobular and glandular tissue [5]. Adipose tissue exhibits both paracrine and endocrine effects. Adipocytes secrete pro- and anti-inflammatory cytokines, peptide hormones that play an important role in maintaining energy homeostasis and regulating the immune system [6]. During the development of obesity, the increased release of adipokines with pro-inflammatory activity (e.g., leptin) and the decrease of those with anti-inflammatory effects (e.g., adiponectin) establishes a chronic inflammatory state that predisposes an individual to the development of metabolic diseases (insulin resistance, type 2 diabetes and cardiovascular disorders) and cancer. Chronic inflammation induced by excessive adipose tissue accumulation causes remodelling of its microenvironment, that is, fibrosis and angiogenesis leading to a pro-oncogenic environment [7]. Obese individuals demonstrate alterations in oestrogen signalling, mainly in the release of aromatase (the enzyme responsible for converting androgens into oestrogens), leading to the formation and progression of oestrogen-dependent tumours [8]. Obesity is further associated with the increased production of reactive oxygen species (ROS), which may contribute to the initiation of breast cancer through damage to cellular DNA [9]. Adipose tissue assumes the role of the primary component of the tumour microenvironment, and the cooperation between adipocytes and tumour cells is being studied by researchers [7].

Leptin regulates foetal development, reproduction, lactation, bone development, haematopoiesis, the immune response, angiogenesis and proliferation of many different cell types, including breast tissue cells [10]. Leptin expression is also regulated by inflammatory mediators and directly correlates with insulin levels and inversely with glucocorticoid levels, and increases in the course of acute infection and sepsis [11]. Elevated leptin levels are thought to be involved in tumour cell growth and invasion through increased expression of vascular endothelial growth factor (VEGF) and by modulating the oestrogen receptor α (ERα), which stimulates aromatase expression and consequently increases oestrogen levels [12,13]. Furthermore, elevated leptin expression in IBrC is thought to be involved in higher tumour grades and increased size [14]. Adiponectin demonstrates the opposite effects with respect to leptin, meaning it shows anti-tumour activities including antiproliferative, antimigratory and proapoptotic [13]. Plasma adiponectin levels are reduced in obese compared to normal weight individuals, negatively correlating with BMI (body mass index) [15]. Hypoadiponectinemia in obesity may result from increased production of pro-inflammatory cytokines, such as tumour necrosis factor α (TNF-α) and interleukin 6 (IL-6), or from negative feedback of adipokine on its own production and the production of its receptors. Low adiponectin levels have been linked to, among other things, an increased risk of developing type 2 diabetes, insulin resistance and various malignancies, including breast cancer [16]. Various studies indicate that in women with hypoadiponectinemia, breast tumours may present a more aggressive phenotype independently of hormone receptor status, for example, by large tumour size, high histological grade and increased angiogenesis and metastasis [13,17]. Studies have analysed that leptin alone or adiponectin alone before treatment have no prognostic values. A different matter is the correlation of these with, for example, BMI [13].

This study is designed to investigate the prognostic value of the pre- and post-treatment serum levels of adiponectin and leptin in luminal A and B IBrC patients based on six-years follow-up. For this study, we used the Kaplan-Meier, logistic and linear regression analyses and the receiver operating characteristic (ROC) curve for estimation of the potential prognostic model’s accuracy in order to establish our preliminary hypothesis.

## 2. Materials and Methods

### 2.1. Study Design

Seventy previously untreated patients with clinically and histological proven primary stage I–II IBrC were enrolled in the study. The subjects were admitted to the Clinical Ward of Breast Cancer and Reconstructive Surgery, Oncology Centre, Prof F. Łukaszczyk Memorial Hospital, Bydgoszcz, Poland between November 2015 and January 2018. All cases were of Polish descent. Weight and height were collected at admission and the BMI was calculated and expressed as kg/m^2^. The median age of patients was 54.5 years old (IQR = 49.0–59.0). Patients received a complete clinicopathological and post-operative examination and were correctly diagnosed according to invasive standards. A classification based on that of the World Health Organization (WHO) was used to classify the histological type of breast cancer. Tumour stage was determined according to the American Joint Committee on Cancer (AJCC; 7th edition).

**Figure 1:**
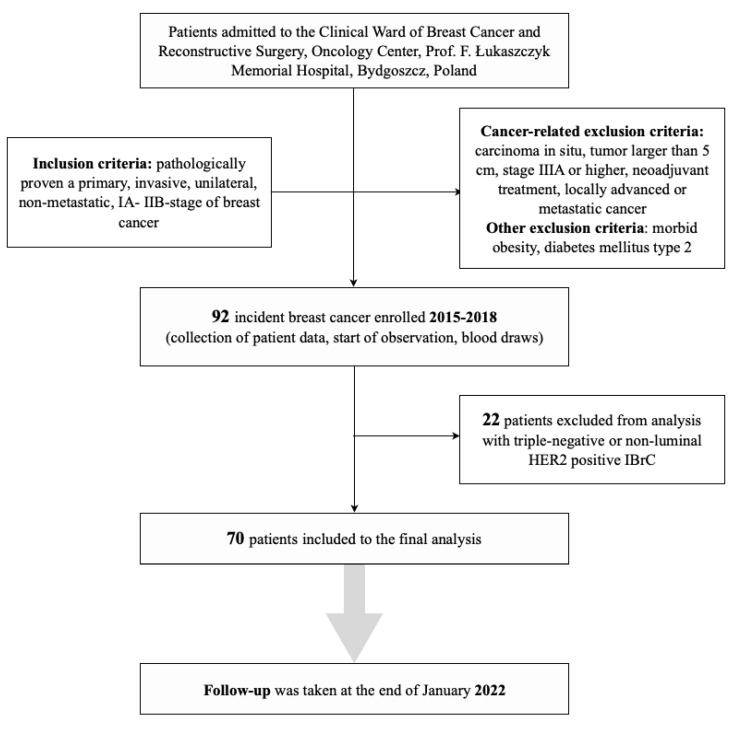
Flowchart of the breast cancer patients selection classification to the study.

The main inclusion criteria were pathologically proven primary, invasive, unilateral, non-metastatic, early stage (IA–IIB) breast cancer. The cancer-related exclusion criteria were as follows: carcinoma in situ, tumour larger than 5 cm, stage IIIA or higher, neoadjuvant treatment, locally advanced or metastatic cancer, triple-negative, non-luminal HER2 positive IBrC. Figure 1 shows the flowchart of patient recruitment for the study. Other exclusion criteria were morbid obesity (BMI over 40 kg/m^2^) and diabetes mellitus type 2.

### 2.2. Treatment Standards

All participants were treated according to standard guidelines established by the National Comprehensive Cancer Network (NCCN) Guidelines for Practice. Fifty-six cases underwent breast-conserving surgery (BCS), seven had a standard mastectomy and seven had a modified radical mastectomy (MRM). All surgical procedures were carried out according to standard conditions. Sixty-eight women received adjuvant treatment. All study participants were treated with surgery as their primary treatment, followed by adjuvant therapy consisting of radiotherapy, brachytherapy, hormone therapy, chemotherapy and immunotherapy. Post-operative radiotherapy was delivered mainly in patients after BCS. In the analysed group, post-operative radiotherapy was administered with X photons with energies of 6/15 MeV and a dose of 42.5 gray (Gy) in 17–20 fractions over 4–6 weeks. Additionally, in half of the women, brachytherapy was applied to the tumour bed at a dose of 10 Gy. Adjuvant chemotherapies were anthracycline-containing (*n* = 23) and non-anthracycline (*n* = 4) containing drugs, given in three to six cycles. The type of the endocrine treatment depended on menopausal status; 40 (57%) used tamoxifen (Egis Pharmaceuticals, Budapest, Hungary), 17 (24%) received aromatase inhibitors (AIs) (Arimidex (anastrozole), AstraZeneca, Cambridge, UK) and seven (10%) were given a combination of tamoxifen and AIs. Four (6%) HER2-positive patients were required to receive an adjuvant immunotherapy (trastuzumab).

### 2.3. Ethical Approval

The study was performed under the appropriate institutional ethics approvals (KB 547/2015) and in accordance with the guidelines of the Declaration of Helsinki. Written informed consent was obtain from each participant.

### 2.4. Follow-Up and Survival Status

Patients were followed from the diagnosis of IBrC to the date of breast cancer recurrence or death or until January 2022, whichever came first. Overall survival (OS) and progression-free survival (PFS) are presented by Kaplan-Meier graphs. The median follow-up was 68.5 months (IQR = 59–72 months). During the analysis, 11 events occurred, one distant metastasis and ten deaths (recurrence rate was 15.71%).

### 2.5. Blood Analysis

Venous blood samples were drawn twice into 4.0 mL tubes (Becton Dickinson Vacutainer^®^, Franklin Lakes, NJ, USA) without anticoagulant in order to determine the concentrations of adiponectin and leptin. The first blood collection occurred 24 h before the surgical procedure (I–pre-treatment values). Collection of the second blood specimen (II–post-treatment values) took place a maximum of three months after the last cytotoxic infusion and generally at nine months (IQR = 6.0–10.0) after the tumour removal procedure to avoid the direct impacts of chemotherapy or surgical wound healing on the levels of adiponectin and leptin.

Material was collected under strict conditions, that is, fasting (after a 12-h fast) and after a 30-min rest. After mixing the samples, they were centrifuged at 3000× *g* at +4° C for 15 min, aliquoted and stored at −80 °C (as specified by the manufacturer) until assayed, but not for more than six months. At the time of the bath analysis, aliquots were limited to one freeze-thaw cycle and storage conditions were maintained.

#### 2.5.1. Serum Leptin Measurement

The baseline serum leptin concentration was measured using a commercially available kit, Human Leptin Enzyme-Linked Immunosorbent Assay (ELISA) Clinical Range (BioVendor Research and Diagnostic products, Brno, Czech Republic; catalogue number: RD191001100), in accordance with the manufacturer’s instructions. The leptin detection limit was 0.2 ng/mL. The intra-assay coefficient of variation (within-run) was 5.9% with an inter-assay coefficient of variation (run-to-run) of 5.6%. The subjects were separated as having low or high values, dichotomised using a cut-off for leptin before treatment of 12.38 ng/mL, based on the median value, and 16.92 ng/mL according to the ROC cut-off for the whole study population. The post-treatment leptin values were as follows: 23.66 ng/mL (median) and 26.88 ng/mL (ROC cut-off).

#### 2.5.2. Serum Adiponectin Analysis

The serum adiponectin level was determined by a human adiponectin ELISA high sensitivity ELISA kit (BioVendor Research and Diagnostic products, Brno, Czech Republic; catalogue number: RD191023100). The adiponectin detection limit was 0.47 ng/mL and had an intra-assay coefficient of variation (within-run) of 3.9% and an inter-assay coefficient of variation (run-to-run) of 6.0%. Patients were divided into having low or high values, dichotomised using a cut-off for pre-treatment adiponectin of 27.2 ng/mL, based on the median value, and 28.49 ng/mL according to the ROC cut-off for the whole study population. The post-treatment adiponectin values were as follows: 32.37 ng/mL (median) and 32.88 ng/mL (ROC cut-off).

### 2.6. Statistical Methods

Statistical analysis was conducted using Statistica version 13.1 (StatStoft^®^, Cracow, Poland). The Shapiro-Wilk test was used to check the normality of the data distribution. Comparisons between two groups of continuous data were performed using the Student’s *t*-test (normal distribution) or the Mann–Whitney test (non-normal distribution). Comparisons between more than two groups of continuous data were performed by univariate ANOVA analysis with normal distributions or the Kruskal–Wallis ANOVA analysis in the case of variables with non-normal distributions. Patient data are presented as mean and standard deviation or median and IQR as appropriate. Leptin data present as median and IQR, while adiponectin data are shown as mean and standard deviation. Furthermore, the data were compared by means of a non-parametric Wilcoxon signed rank for two dependent variables. To assess relations between the studied variables, a correlation analysis was performed. The Spearman’s rank order correlation test was used to test the correlations between the studied parameters. The ROC curves, area under a curve (AUC) and Youden’s index were also used in the analysis. Optimal cut-off values were defined. Kaplan-Meier curves were used to express survival times and the long-rank test was used to compare survival times. The term “overall survival” (OS) describes the period of time between randomization or treatment beginning and death. Progression-free survival (PFS) refers to the time from randomization or initiation of treatment to the occurrence of disease progression or death. Leptin and adiponectin were also included in multiple linear regression models with adjustments for BMI, age, parity, menopausal status, smoking status, tumor stage, tumor diameter, histological type, intrinsic type and nodal involvement. The statistical significance was predetermined as *p* < 0.05.

## 3. Results

### 3.1. Baseline Characteristics

Seventy women were selected who met the inclusion and exclusion criteria for the study. Table 1 present the baseline characteristic of study group. The median age was 54.5 years (IQR = 49.0–59.0), median BMI was 25.06 kg/m^2^ (IQR = 22.6–28.8) and median tumour size was 1.5 cm (IQR = 1.1–2.1). Of the 70 women, 26 were premenopausal and 44 postmenopausal. In the TNM classification of breast cancer, there were 48 patients at stage T1 and 22 patients at T2. Analysing the histological type of breast cancer, 61 patients had invasive ductal carcinoma and nine had invasive lobular carcinoma. Only 17 of the 70 patients had metastases to local lymph nodes. Considering the molecular type of breast cancer, 50 patients had luminal A cancer, 16 had luminal B HER (negative) and four had luminal B HER (positive) cancer. Sixty-five patients were progesterone receptor and E-cadherin positive. The Ki-67 proliferation index was below 20% in 50 patients. Fifty-six patients underwent BCS and 14 had a mastectomy. The breast cancer of 43 patients did not require chemotherapy and 68 women received endocrine therapy. During the observation (the median follow-up was 68.5 months [IQR = 59–72 months]), 11 events occurred, one distant metastasis and ten deaths. The recurrence rate was 15.71%.

### 3.2. Leptin Levels before and after Treatment with Respect to Clinical Parameters

Table 2 demonstrates the clinicopathological features with respect to pre- and post-treatment leptin concentrations. Generally, post-treatment leptin concentrations were higher independently of clinicopathological factors with respect to pre-treatment leptin levels. Significantly higher levels of pre-treatment leptin concentrations in overweight and obese patients (*p* < 0.0001) were noted. In addition, with respect to the histological types of tumour, pre- and post-treatment leptin concentrations showed a tendency to significance. Both pre- and post-treatment concentrations were higher for invasive ductal carcinoma (*p* = 0.0595 and *p* = 0.0729, respectively) than for invasive lobular carcinoma. Additionally, a significantly higher post-treatment leptin concentration was observed in E-cadherin positive tumours (*p* = 0.0350). Pre-treatment leptin concentrations were higher in E-cadherin positive breast cancers with a tendency to significance (*p* = 0.0611).

Table 3 and Table 4 present leptin concentrations with regard to the types of therapy. Regardless of the treatment pattern, leptin concentration increased after treatment application. 

### 3.3. Clinical Presentation of Patients in Relation to Pre- and Post-Treatment Adiponectin Concentrations

The next step in the statistical analysis (Table 5) was to evaluate the clinical parameters in relation to pre- and post-treatment adiponectin concentrations. Generally, post-treatment adiponectin concentrations were higher independently of clinicopathological factors. Lower concentrations were noted in overweight and obese patients than in patients with normal BMI in relation to pre- and post-treatment adiponectin concentrations (*p* = 0.009 and *p* = 0.0002, respectively). Higher pre-treatment adiponectin levels were observed in patients without children or with 1–2 children than in women who had given birth three or more times (*p* = 0.0385). In addition, higher post-treatment adiponectin concentrations were noted in patients with a negative E-cadherin tumour (*p* = 0.0202). A similar increasing effect of pre-treatment adiponectin concentration was observed with a tendency to significance (*p* = 0.0775). 

Additional analysis was performed to evaluate the adiponectin concentrations with regard to the types of therapy (Tables S1 and S2). Breast-conserving therapy was associated with increased adiponectin level (*p* = 0.0043), similar results were obtained with respect to chemotherapy based on anthracykline (*p* = 0.0043). Tamoxifen demonstrates similar effect on adiponectin level (*p* = 0.0076). Application of combine therapy with chemotherapy was associated with elevation of adiponectin concentration (*p* = 0.0019).

### 3.4. Relationships between Clinicopathological Features and between Adipokines Concentrations

**Figure 2:**
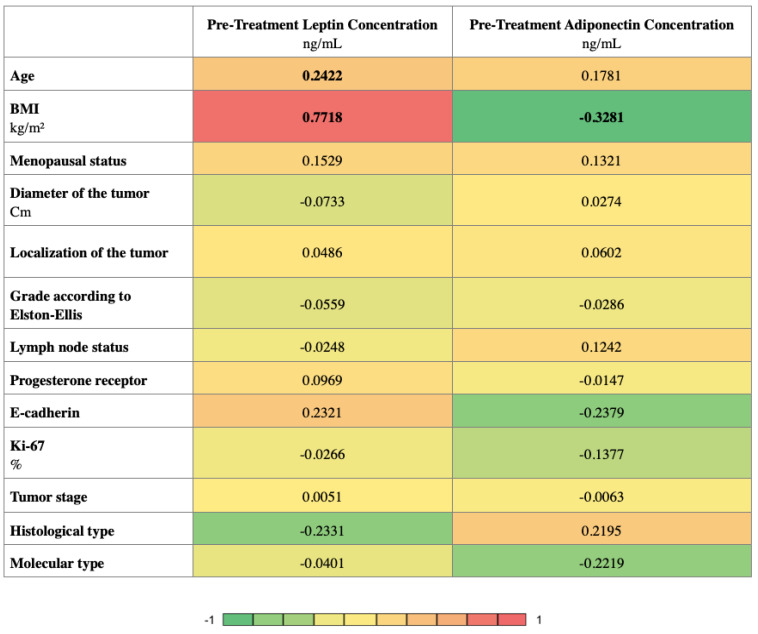
Heatmap displaying the r values obtained from Spearman correlation analysis performed among clinicopathological features; *p*-values < 0.05 were considered to indicate statistical significance and are marked in bold. BMI: Body Mass Index; Ki67: marker of proliferation Ki-67.

Figure 2 presents the correlation analysis which was performed to find relationships between pre-treatment adipokines and the clinical variables. The analysis was carried out using the Spearman’s rank correlation and is presented in the form of a heatmap. As a result, the pre-treatment leptin concentration was found to correlate positively with age and BMI (r = 0.2422, r = 0.7718, respectively). The pre-treatment adiponectin concentration correlates negatively with BMI (r = −0.3281).

**Figure 3:**
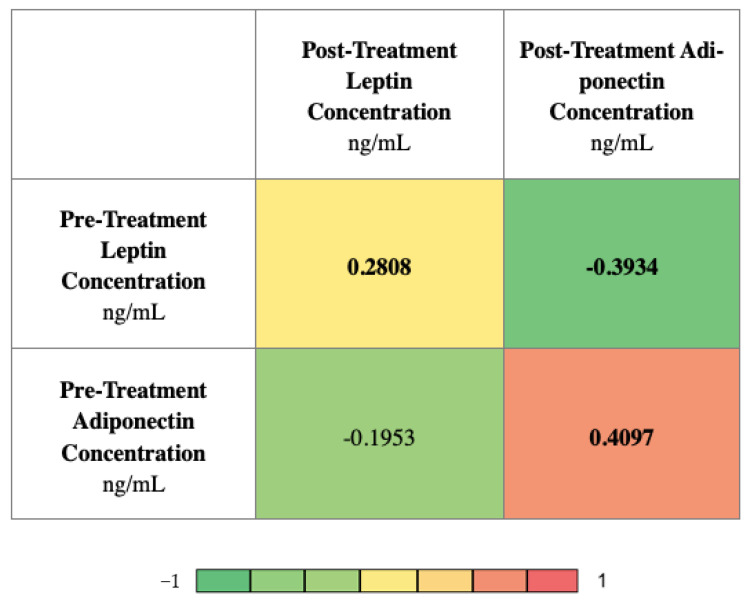
Heatmap displaying the r values obtained from Spearman correlation analysis performed among investigated adipokines concentrations; *p*-values < 0.05 were considered to indicate statistical significance and are marked in bold.

Figure 3 presents the correlations between the investigated adipokine concentrations. The analysis was carried out using the Spearman’s rank correlation and is presented in the form of a heatmap. The pre-treatment leptin concentration correlates positively with the post-treatment leptin concentration (r = 0.2808) and negatively with the post-treatment adiponectin concentration (r = −0.3934). The pre-treatment adiponectin concentration correlates positively with the post-treatment adiponectin concentration (r = 0.4097).

### 3.5. Receiver Operating Characteristic (ROC) Curve Analysis of Adipokines before Treatment

**Figure 4:**
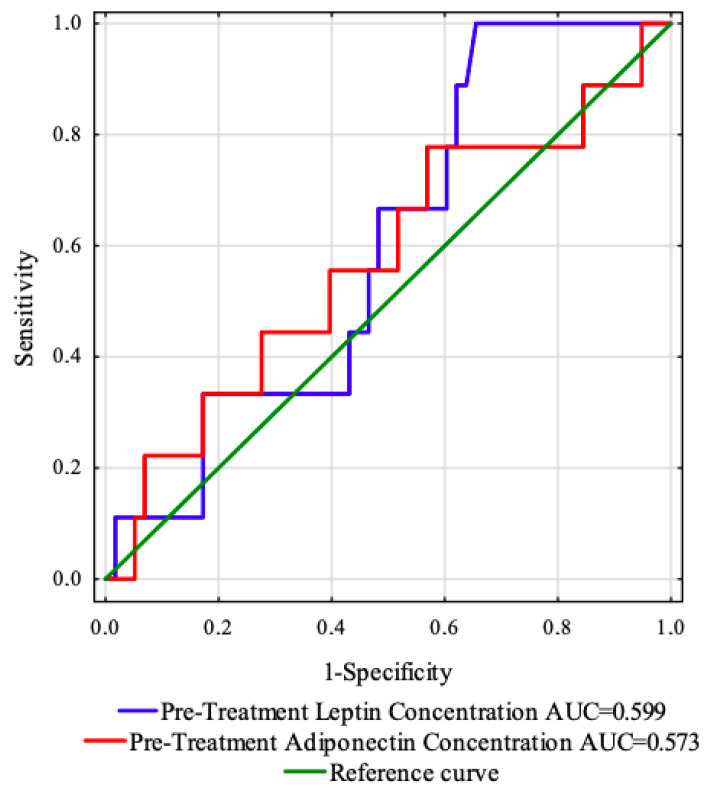
ROC curves and AUC values of the investigated adipokines before treatment.

The ROC curve was created to evaluate the predictive value of the studied adipokines concentrations (before treatment) to predict the OS and PFS (Figure 4). The areas under the curve with 95% confidence interval were established (AUC, 95% thresholds with sensitivity and specificity). For this analysis, the parameters showed no prognostic value in our cohort (Table 6).

### 3.6. Survival Analysis Regarding Pre-Treatment Adipokines

The next step in the statistical analysis was to use the cut-off points from the ROC curve for leptin and adiponectin before treatment and to calculate the cut-off point based on the median for these adipokines (Table 7). The patients were then divided into two groups: below and above the cut-off point. The OS and PFS were assessed for each group. The median follow-up was 68.5 months (IQR = 59–72 months). During the observation, ten patients died due to systemic metastatic disease and only one case had a recurrence. The recurrence rate was 15.71%.

**Figure 5:**
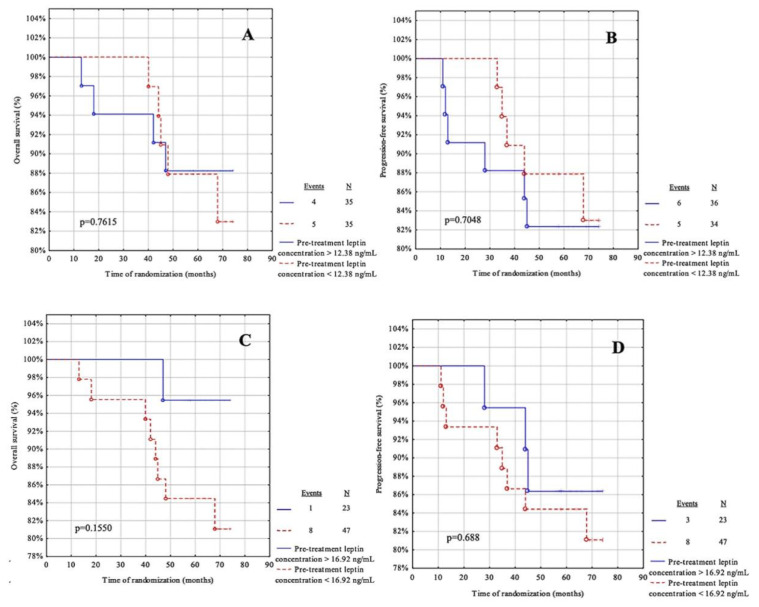
Kaplan-Meier curves for the overall survival (OS) and progression-free survival (PFS) analysis regarding (**A**,**B**) pre-treatment leptin concentration according to median value cut-off; (**C**,**D**) pre-treatment leptin concentration according to ROC cut-off. Significant differences are denoted by bold *p*-values.

**Figure 6:**
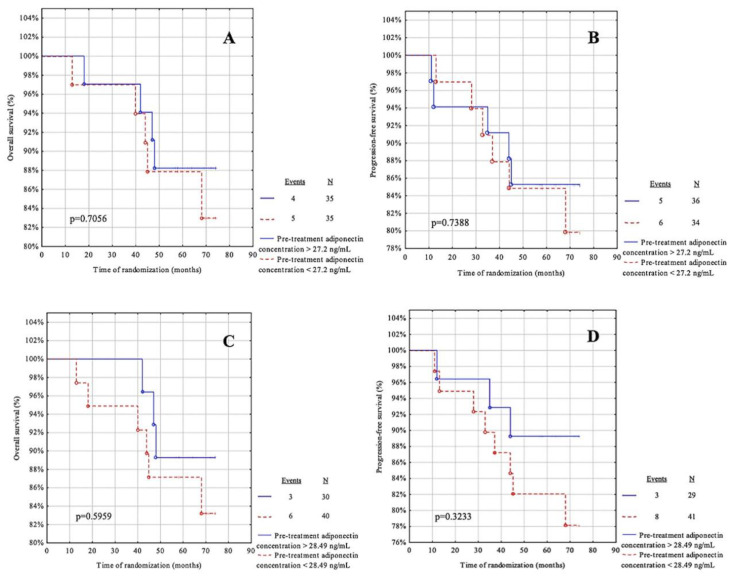
Kaplan-Meier curves for the overall survival (OS) and progression-free survival (PFS) analysis regarding (**A**,**B**) pre-treatment adiponectin concentration according to median value cut-off; (**C**,**D**) pre-treatment adiponectin concentration according to ROC cut-off. Significant differences are marked with a bold *p*-values.

For this analysis, the parameters showed no predictive value in our cohort (Figure 5A–D and Figure 6A–D).

### 3.7. Receiver Operating Characteristic (ROC) Curve Analysis of Adipokines after Treatment

**Figure 7:**
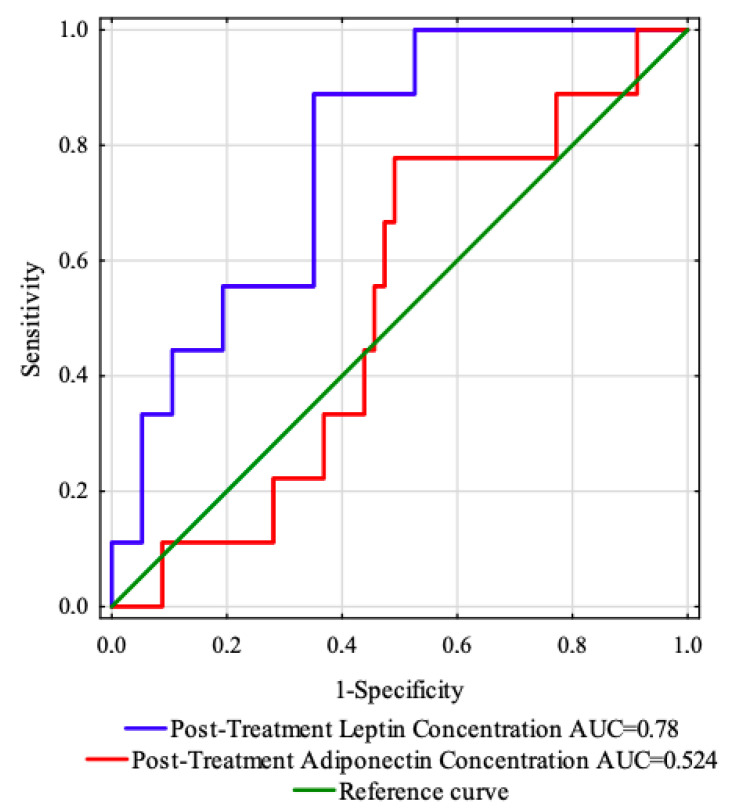
ROC curves and AUC values of the investigated adipokines after treatment.

Figure 7 shows the ROC curve determined to estimate the prognostic value of the concentrations of the examined adipokines (after treatment) for predicting the OS and PFS. The areas under the curve with 95% confidence intervals (AUC, 95% thresholds with sensitivity and specificity) were established.

Following the results of the study, the post-treatment leptin concentration was found to be the strongest predictor of disease relapse (AUC = 0.78, *p* < 0.0001). Using the maximum value of the Youden index, a post-treatment leptin concentration of 26.88 ng/mL with sensitivity of 88.9% and specificity of 64.9% was identified as the best cut-off value to distinguish patients with or without disease recurrence (Table 8).

### 3.8. Survival Analysis Regarding Post-Treatment Adipokines

Following this, the cut-off points from the ROC curve were used, and the cut-off points were calculated based on the median for these adipokines (Table 9). Patients were divided into two groups: below and above the cut-off point. The OS and PFS were assessed for each group. The median follow-up was 68.5 months (IQR = 59–72 months). During follow-up, ten patients died due to systemic metastases and only one patient had a relapse. The recurrence rate was 15.71%.

**Figure 8:**
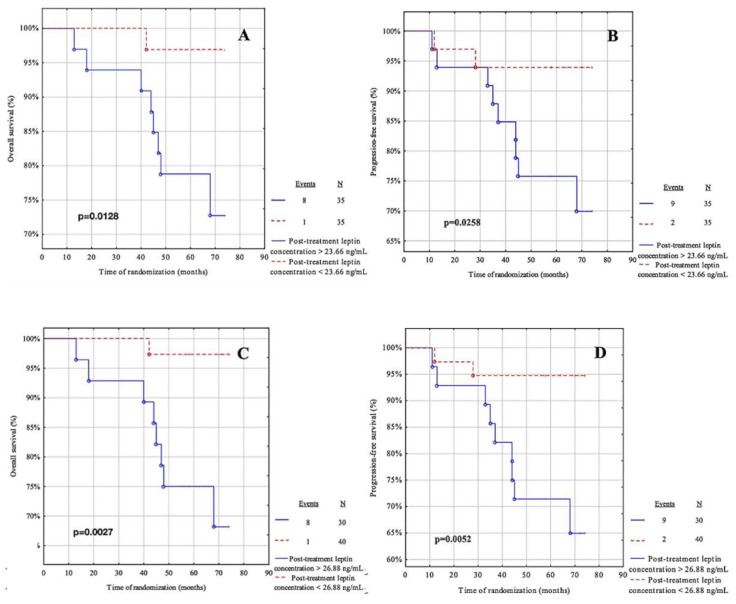
Kaplan-Meier curves for the overall survival (OS) and progression-free survival (PFS) analysis regarding (**A**,**B**) post-treatment leptin concentration according to median value cut-off; (**C**,**D**) post-treatment leptin concentration according to ROC cut-off Significant differences are marked with a bold *p*-values.

Patients with post-treatment leptin concentrations below 23.66 ng/mL had a significantly better OS than those with post-treatment leptin concentrations above 23.66 ng/mL (*p* = 0.0128) (Figure 8A). Thirty-five patients had concentrations below 23.66 ng/mL and 35 had concentrations above 23.66 ng/mL. There was one (2.86%) event in the group of women with concentrations below 23.66 ng/mL and eight (22.86%) relapses in the group with concentrations above 23.66 ng/mL. Similar results were obtained for the post-treatment leptin concentrations (Figure 8C) with the cut-off point determined from the ROC curve (*p* = 0.0027).

Subjects with post-treatment leptin levels below 23.66 ng/mL had a significantly better PFS than patients with post-treatment leptin levels above 23.66 ng/mL (*p* = 0.0128) (Figure 8B). Thirty-five patients had concentrations below 23.66 ng/mL and 35 had concentrations above 23.66 ng/mL. There were two (5.71%) occurrences in the group of women with concentrations below 23.66 ng/mL and nine (25.71%) relapses in the group with concentrations above 23.66 ng/mL. Similar outcomes were obtained for the post-treatment leptin concentrations (Figure 8D) with the cut-off point determined from the ROC curve (*p* = 0.0052).

**Figure 9:**
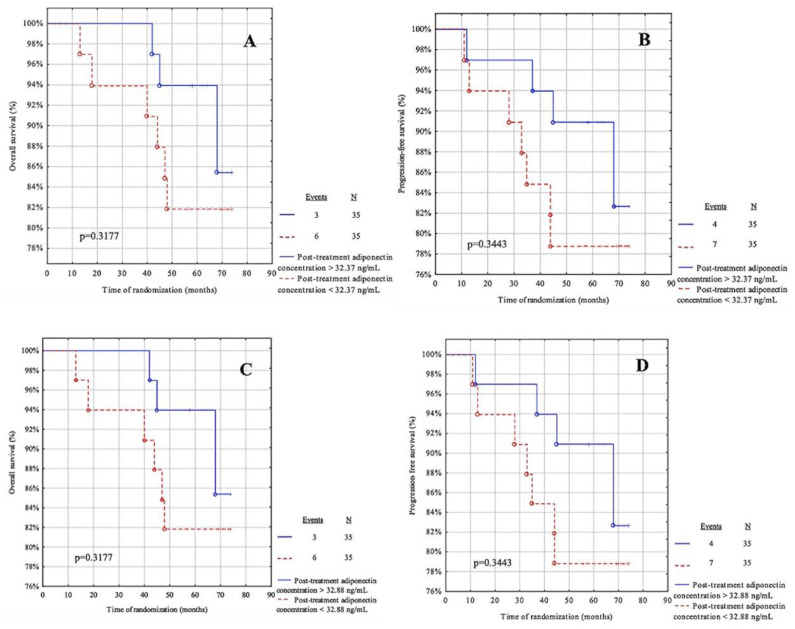
Kaplan-Meier curves for the overall survival (OS) and progression-free survival (PFS) analysis regarding (**A**,**B**) post-treatment adiponectin concentration according to median value cut-off; (**C**,**D**) post-treatment adiponectin concentration according to ROC cut-off. Significant differences are marked with a bold *p*-values.

Figure 9A,B present the post-treatment adiponectin concentrations according to the median cut-off, while Figure 9C,D present the concentrations according to the cut-off from the ROC curve. For this analysis of the post-treatment adiponectin, these parameters showed no predictive value in our cohort.

### 3.9. Association of Adipokines with Disease-Free Survival in Linear Regression

The last step in the statistical analysis (Table 10) was to determine linear regression models between pre- and post-treatment adipokines and disease-free survival. Breast cancer recurrence was negatively associated only with post-treatment leptin concentration. Model 1, 2 and 3 independently of adjusted factors showed that the higher post-treatment leptin concentration was correlated with a shorter disease-free survival (*p* = 0.0078; *p* = 0.0031, *p* = 0.0324, respectively). Adjusting for age, BMI, parity, menopausal status, smoking status, tumour stage, tumour diameters, intrinsic type, histological type and nodal involvement, Model 4 had a tendency to significance (*p* = 0.0502) in similar manner.

## 4. Discussion

Breast cancer is the most common malignancy in women worldwide. Its origin is based on processes of proliferation, evasion of growth suppressors, abnormalities in cell death, initiation of invasion and metastasis, disruption of cellular homeostasis and evasion of immune destruction with the additional important possibility of recurrence [13]. One of the risk factors for developing breast cancer, confirmed in many studies, is obesity. As an endocrine organ, adipose tissue produces a large range of factors that can influence the development of breast cancer. Leptin and adiponectin are the two main hormones secreted by adipocytes. The pro-cancerogenic effects of leptin and, conversely, the anti-cancerogenic effects of adiponectin are due to the modulation of the signalling pathways involved in proliferation and the subtle regulation of the apoptotic response [18].

### 4.1. Pre- and Post-Treatment Leptin and Adiponectin Values in Relation to Demographic, Anthropometric and Clinicopathological Characteristics

In the first stage of our study, we presented clinicopathological features with respect to pre- and post-treatment leptin concentrations. We observed significantly higher pre-treatment leptin concentrations in overweight and obese patients. These results are confirmed by the Spearman’s correlation in which pre-treatment leptin concentration correlates positively with BMI. Bhat et al. revealed results that appear consistent with ours. They showed that leptin concentrations were higher in obese individuals and had a direct correlation with the degree of obesity [19]. Interestingly, in their study, Pan et al. divided patients by BMI level and found that serum leptin levels were not associated with breast cancer in healthy normal-weight individuals or normal-weight individuals with breast cancer. They noted a strong association between breast cancer risk and higher leptin levels in overweight or obese patients [20]. Furthermore, in our research we observed a tendency to significantly higher pre- and post-treatment leptin concentrations in invasive ductal carcinoma. Our results seem to support those of Karacay et al. who observed a higher rate of leptin receptor in invasive ductal carcinoma. According to them, leptin may have an autocrine promoting effect on breast cancer carcinogenesis and its inhibition may be effective in the treatment of breast cancer [21]. The study by Révillion et al. supports the conclusions formulated by Karacay et al. regarding the autocrine action of leptin on breast cancer cells. They showed that practically all breast cancer samples studied co-expressed the two main leptin receptor isoforms; long form leptin receptor (OBR-L) and short form leptin receptor (OBR-S). However, Révillion et al. showed increased leptin expression in patients with lobular carcinoma compared to other tumour types. [22]. Interestingly, in our study, we showed that E-cadherin positive tumours are characterised by elevated leptin concentrations after treatment. The pre-treatment leptin concentrations were close to being statistically significant. These results confirm the researchers’ findings. Mauro et al. investigated the mechanism of tumour growth and showed that leptin increased E-cadherin-dependent cell-cell adhesion. This implies that leptin is able to promote tumour cell proliferation and adhesion by increasing E-cadherin expression. This may indicate an important role for leptin in stimulating the growth and progression of primary breast tumour cells, especially in obese women [23]. In the next stage of the research, we compared leptin concentrations before and after treatment according to the types of applied treatment. Higher pre-treatment leptin levels were observed with a trend towards significance in patients treated with non-anthracycline chemotherapy or no chemotherapy. In addition, higher pre-treatment leptin levels were reported with a trend towards significance in patients whose doctor used combination therapy with hormone therapy. Interestingly, some studies indicate that leptin appears to stimulate ligand-independent oestrogen-receptor activation via the up-regulation mediator subunit 1 (Med1). It has been shown that Med1 may contribute to tamoxifen resistance in breast cancer cells, and high Med1 expression correlates with poor prognosis in patients treated with tamoxifen [24,25]. In the following stage of the study, the Spearman’s correlation analysis showed that leptin before treatment correlates positively with age. We speculate that this may be due to a decrease in muscle mass versus an increase in body fat and a change in plasma concentrations of many hormones with age. However, the researchers’ studies do not confirm our results. In their study, Isidori et al. showed an inverse relationship between age and leptin concentration [26].

In the current study, adiponectin concentrations (before and after treatment) were compared in relation to clinical parameters. In our research we observed lower concentrations of pre- and post-treatment adiponectin levels in obese women. These results are confirmed by Spearman’s correlation in which the pre-treatment adiponectin concentration correlates negatively with BMI. Our results seem to support those of Matsubara et al. who observed decreased adiponectin concentrations in women with the highest BMI tertile compared with those in the middle and lowest BMI tertiles [27]. Furthermore, in our study, we observed higher pre-treatment adiponectin concentrations in non-pregnant women or those who had given birth to 1–2 children. Due to the fact that adiponectin is an adipokine with anti-inflammatory and anti-cancerogenic effects, we can speculate that this may have a protective effect. The present study also showed that in patients with E-cadherin negative tumours, we observed higher post-treatment adiponectin levels. Furthermore, a similar effect was demonstrated with a tendency to significance in pre-treatment adiponectin concentrations.

### 4.2. Post-Treatment Leptin Concentration as a Predictor of Relapse

The ROC curve and AUC value revealed that post-treatment leptin concentration is the best predictor of disease relapse. (AUC = 0.78, *p* < 0.0001). According on the Youden index, we indicated that the cut-off point of 26.88 ng/mL for the leptin concentration after treatment may serve as a value that distinguishes patients with or without disease recurrence, with a sensitivity of 88.9% and specificity of 64.9%. Furthermore, according to the Kaplan-Meier curves, we suggest that a post-treatment leptin concentration below 23.66 ng/mL has better OS and PFS. There was one event in the group of women with concentrations below 23.66 ng/mL and eight relapses in the group with concentrations above 23.66 ng/mL considering the OS analysis. Taking into account the PFS, there were two events in the group of women with concentrations below 23.66 ng/mL and nine recurrences in the group with concentrations above 23.66 ng/mL. Similar results were obtained for the post-treatment leptin concentrations with the ROC cut-off point. The recurrence rate was 15.71%. Using the linear regression models, it was ultimately found that regardless of the model used, excluding tumour-dependent factors and baseline factors (such as age, BMI, parity, menopausal status, smoking status, tumour stage, tumour diameters, intrinsic type, histological type, nodal involvement), higher post-treatment leptin concentration is correlated with shorter disease-free survival. Our results seem to support those of Cho et al. who observed a positive association between leptin and breast cancer recurrence, but only in patients with hormone receptor-positive tumours. Leptin, whose expression is elevated in overweight/obese individuals, promotes the production of oestrogen as a result of increased aromatase activity which may be related to the progression of oestrogen receptor-positive cancers [28]. On the contrary, Obi et al. did not find supporting evidence for an association between leptin levels and overall mortality [29]. We want to make reference to the findings of an earlier study. [13]. Bielawski et al. noted increased recurrence rates and cancer-specific mortality in cases with a normal BMI and the highest concentration of leptin. Furthermore, the pre-treatment leptin concentration showed no predictive value as a single biomarker. Comparing our current results with those of the previous study, it seems appropriate to analyse leptin concentrations before and after treatment and to carry out longer follow-ups. Elevated leptin levels after treatment and consequently increased recurrence and mortality rates in breast cancer patients may be due to resistance to chemotherapy. One mechanism responsible for this is the nuclear factor kappa-light-chain-enhancer of activated B cells (NFκB) signalling which controls the DNA transcription of several genes. Leptin stimulates NFκB, which may improve the survival of chemotherapy-treated cancer cells [30]. Another mechanism is hypoxia inducible factor (HIF), which correlates with the activation of leptin signalling in many malignancies, including endometrial, pancreatic, breast and colon cancers. Hypoxia in cancer is associated with poor outcomes and chemoresistance [31,32]. Letrozole resistance in IBrC has been shown by Pang et al. to be conferred by the leptin/PBX3/FGFR1 cascade. In a signal transducer and activator of transcription 3 (STAT3) -dependent mechanism, hyperleptinemia increases pre-B-cell leukaemia homeobox transcription factor 3 (PBX3) expression. By bringing the metastasis-associated 1-histone deacetylase 2 (MTA1-HDAC2) complex straight to the fibroblast growth factor receptor 1 (FGFR1) promoter, PBX3 increases the trans-activation of FGFR1. In the end, the resulting FGFR1 amplification impairs the therapy response to letrozole [33]. Gu et al. investigated the potential mechanisms by which high leptin expression reduces the sensitivity of ovarian cancer cells to treatment. Ovarian cancer patients were treated with paclitaxel (PTX) chemotherapy and the leptin mRNA expression data were analysed. They showed that the highest scoring was the epithelial-to-mesenchymal transition (EMT) gene set. EMT is the biological process by which polarised epithelial cells transform into mesenchymal cells. This process is not only common in tumour-initiating cells (increasing their invasive and migratory capacity), but is also closely associated with multiple drug resistance in human tumours [34].

### 4.3. Strengths and Limitation of the Study

The main limitations of the study were the small sample size and the fact that we recruited patients from only one study centre, which in itself limits the number of patients. Getting patients’ permission to participate and having them meet very tight inclusion criteria determined the sample size. In addition, we recruited patients of Polish origin only, so our results may not be applicable to other ethnic groups. We recruited patients at an early stage of breast cancer without metastases, so we cannot determine what the prognostic value would be for larger and more advanced tumours. The strength of this study is that it was based on a six-year follow-up of patients with complete clinicopathological characteristics. All blood samples were taken prospectively from fasting patients one day prior to surgery, reducing any variations that would have happened if the samples had been taken at various times.

## 5. Conclusions

Despite the limited series of luminal IBrC patients included to the study, our results suggest a few important points: (1) Adjuvant IBrC therapy most likely increased the leptin and adiponectin levels, regardless of treatment patterns; (2) It seems that only post-treatment leptin concentration was associated with the future outcomes of luminal IBrC patients, since a concentration of leptin higher than 26.88 ng/mL has been shown to promote the probability of recurrence and morbi-mortality in the IBrC cohort; (3) Pre-treatment leptin levels correlated positively with leptin concentrations and negatively with adiponectin levels after adjuvant IBrC therapy, thus the pre-operative adipokines profile may reflect its future associations; (4) Regardless of the linear regression model used, only post-treatment leptin levels showed prognostic implications; (5) Further study is necessary to determine whether chemotherapy resistance is the cause of elevated post-treatment leptin levels, increased rates of recurrence and mortality in patients with breast cancer.

## Figures and Tables

**Table 1:** Baseline and clinical characteristics of the study group.

Demographic and Clinical Data	Number of Patients (%)
Age	
<55 years	35 (50%)
>55 years	35 (50%)
Menopausal status	
pre-menopausal	26 (37%)
post-menopausal	44 (63%)
BMI (kg/m^2^)	
Normal (18.5 ≤ 24.99)	34 (48.6%)
Overweight (25 ≤ 29.99)	23 (32.8%)
Obese (30 ≤ 39.99)	13 (18.6%)
Parity status	
0	6 (9%)
1–2	50 (71%)
3 and more	14 (20%)
Localization of tumour	
Right breast	36 (51%)
Left breast	34 (49%)
Diameter of the tumour	
<2 cm	48 (69%)
>2 cm <5 cm	22 (31%)
Lymph node status	
N0	53 (76%)
N1	17 (24%)
Histological type	
IDC	61 (87%)
ILC	9 (13%)
TNM staging classification	
T1	48 (69%)
T2	22 (31%)
Grade according to Elston-Ellis	
1 + 2	61 (87%)
3	9 (13%)
Molecular type	
Luminal A (HR+/HER2−/Ki-67 < 20%)	50 (71%)
Luminal B (HR+/HER2−/Ki-67 ≥ 20%)	16 (23%)
Luminal B HER2+ (HR+ HER2+)	4 (6%)
Staging	
I	35 (50%)
II	35 (50%)
Progesterone receptor (PR)	
Negative	5 (7%)
Positive	65 (93%)
E-cadherin	
Negative	5 (7%)
Positive	65 (93%)
Ki-67	
<20%	50 (71%)
≥20 %	20 (29%)

BMI: body mass index; N0: no evidence of spread to lymph nodes; N1: spread to auxiliary lymph nodes; IDC: invasive ductal carcinoma; ILC: invasive lobular carcinoma; T1: tumour is smaller than 2 cm across; T2: tumour is 2 to 5 cm across; HR+: hormone receptor positive; HER2-: human epidermal growth factor receptor 2 negative; HER2+: human epidermal growth factor receptor 2 positive; Ki67: marker of proliferation Ki-67.

**Table 2:** Leptin concentrations with respect to clinicopathological features.

Analyzed Parameters/ Number of Patients	Pre-Treatment Leptin Concentration (ng/mL)	Post-Treatment Leptin Concentration (ng/mL)	*p*-Value
Age <55 years 35 ≥55 years 35	*p* = 0.1130 8.43 4.00/16.92 12.55 7.67/20.78	*p* = 0.6956 21.17 13.02/34.39 25.07 14.17/32.97	**0.0003** **0.0025**
BMI (kg/m^2^) Normal (18.5 ≤ 24.99) 34 Overweight (25 ≤ 29.99) 23 Obese (30 ≤ 39.99) 13	***p* < 0.0001** 6.11 3.12/9.70 14.79 9.60/20.64 25.26 16.92/30.93	*p* = 0.2419 16.99 9.56/32.97 24.23 16.54/32.17 26.88 19.50/42.95	**0.0001** **0.0051** 0.3824
Menopausal status Premenopausal 26 Postmenopausal 44	*p* = 0.2166 6.99 3.87/18.60 12.58 6.73/19.40	*p* = 0.9416 23.51 14.06/37.45 23.93 12.94/32.61	**0.0019** **0.0005**
Parity status 0 6 1–2 50 3 and more 14	*p* = 0.8361 7.84 5.57/14.41 12.55 5.69/19.31 12.38 4.92/16.92	*p* = 0.1044 15.67 9.56/19.23 22.94 12.41/32.17 29.83 21.37/39.42	0.3454 **0.0011** **0.0015**
Postmenopausal hormone therapy No 59 Yes 11	*p* = 0.6971 12.45 5.13/19.31 7.67 5.26/15.68	*p* = 0.1947 25.36 13.62/38.30 18.76 13.23/21.87	**<0.0001** 0.5754
Localization of the tumour Right breast 36 Left breast 34	*p* = 0.6974 12.04 5.57/17.89 12.38 5.26/19.21	*p* = 0.6874 25.06 13.62/38.30 22.08 13.56/32.79	**0.0001** **0.0086**
Diameter of the tumour <2 cm 48 ≥2 cm < 5 cm 22	*p* = 0.3855 9.60 4.60/19.40 13.57 7.79/17.89	*p* = 0.9187 24.50 14.34/32.79 21.30 10.08/36.60	**<0.0001** **0.0186**
Lymph node status N0 53 N1 17	*p* = 0.8458 11.89 5.34/19.21 12.63 4.60/16.92	*p* = 0.1854 20.46 12.94/32.17 28.56 18.86/45.77	**0.0007** **0.0005**
TNM staging classification T1 48 T2 22	*p* = 0.3818 9.60 4.60/19.40 13.57 7.79/17.89	*p* = 0.9133 24.50 14.34/32.79 21.30 10.08/36.60	**<0.0001** **0.0186**
Grade according to Elston-Ellis 1 + 2 61 3 9	*p* = 0.6495 12.38 5.57/19.21 10.07 3.61/20.40	*p* = 0.1254 20.46 12.94/32.61 31.25 26.58/40.63	**<0.0001** **0.0281**
Histological type Invasive ductal carcinoma (IDC) 61 Invasive lobular carcinoma (ILC) 9	*p* = 0.0595 12.61 6.11/19.40 5.57 2.83/11.56	*p* = 0.0729 24.77 14.50/36.60 10.08 6.87/25.35	**<0.0001** 0.0858
Molecular type Luminal A (HR+/HER2-/Ki-67 < 20%) 50 Luminal B (HR+/HER2-/Ki-67 ≥ 20%) 16 Luminal B HER2 + (HR+ HER2+) 4	*p* = 0.9479 12.55 5.46/18.69 9.70 4.00/19.21 12.85 4.75/20.40	*p* = 0.7267 23.93 14.49/32.97 21.73 9.22/33.86 20.40 7.31/35.67	**0.0001** **0.0409** 0.0679
Staging I 35 II 35	*p* = 0.7299 9.19 4.85/20.71 12.63 6.17/17.89	*p* = 0.3657 20.46 13.90/31.00 25.54 12.94/42.95	**0.0055** **0.0001**
Progesterone receptor Negative 5 Positive 65	*p* = 0.4381 9.50 7.79/9.70 12.55 5.26/19.40	*p* = 0.1460 43.02 27.41/51.50 22.79 13.62/32.17	**0.0431** <**0.0001**
E-cadherin Negative 5 Positive 65	*p = *0.0611 3.12 2.83/4.43 12.55 6.11/19.21	***p* = 0.0350** 8.74 5.91/10.08 24.77 14.50/32.97	0.3452 **<0.0001**
Ki-67 (%) <20 50 ≥20 20	*p* = 0.4450 12.21 4.92/17.89 13.40 6.17/23.88	*p* = 0.6204 20.46 13.62/32.61 26.12 14.48/38.01	**0.0002** **0.0038**

Data are expressed as median (Me) and inter-quartile range (IQR); *p*-values < 0.05 were considered to indicate statistical significance and are marked in bold. BMI: body mass index; N0: no evidence of spread to lymph nodes; N1: spread to axillary lymph nodes; T1: tumour is smaller than 2 cm across; T2: tumour is 2 to 5 cm across; HR+: hormone receptor positive; HER2-: human epidermal growth factor receptor 2 negative; HER2+: human epidermal growth factor receptor 2 positive; Ki67: marker of proliferation Ki-67.

**Table 3:** Leptin concentrations according to the types of surgery and adjuvant therapy in IBrC subjects.

Feature/ Number of Patients	Pre-Treatment Leptin Concentration (ng/mL)	Post-Treatment Leptin Concentration (ng/mL)	*p*-Value
Surgery BCS + Radiotherapy − BCT 56 Mastectomy 14	*p* = 0.1058 9.55 3.06/14.87 12.63 6.11/20.64	*p* = 0.9101 23.09 10.92/36.6 24.23 14.17/32.61	**0.0019** **0.0002**
Chemotherapy Anthracykline 23 Non-anthracycline 4 No 43	*p* = 0.0967 8.11 3.56/15.90 14.43 4.35/28.24 13.53 6.11/20.64	*p* = 0.6341 24.77 9.69/38.30 31.25 19.28/40.44 22.79 14.50/32.17	**0.0006** 0.1441 **0.0019**
Endocrine therapy Tamoxifen 40 Inhibitor aromatase 17 Tamoxifen and inhibitor aromatase 7 Other type 4 No 2	*p* = 0.5685 13.08 6.14/19.93 11.56 4.92/17.89 14.79 4.60/23.60 3.93 2.59/5.26 8.55 2.68/14.41	*p* = 0.2121 19.52 11.05/31.78 25.73 14.49/41.43 32.61 22.79/49.50 23.06 13.73/30.34 17.44 15.65/19.23	**0.0319** **0.0012** **0.0180** 0.1797 0.1797

Data are expressed as median (Me) and inter-quartile range (IQR); *p*-values < 0.05 were considered to indicate statistical significance and are marked in bold. BCS: breast-conserving surgery; BCT: breast-conserving therapy.

**Table 4:** Leptin concentrations regarding types of therapy in IBrC patients.

Feature/ Number of Patients	Pre-Treatment Leptin Concentration (ng/mL)	Post-Treatment Leptin Concentration (ng/mL)	*p*-Value
Monotherapy	*p* = 0.0986	*p* = 0.7621	
2	8.55	17.44	0.1797
Combination therapies with	2.68/14.41	15.65/19.23	
chemotherapy	8.11	25.35	**0.0002**
26	3.72/16.92	9.69/38.30	
Combination therapies with hormone therapy	13.53	23.09	
42	6.73/20.64	14.49/32.61	**0.0030**

Data are expressed as median (Me) and inter-quartile range (IQR); *p*-values < 0.05 were considered to indicate statistical significance and are marked in bold.

**Table 5:** Adiponectin concentrations with respect to clinicopathological features.

Analyzed Parameters/ Number of Patients	Pre-Treatment Adiponectin Concentration (ng/mL)	Post-Treatment Adiponectin Concentration (ng/mL)	*p*-Value
Age <55 years 35 ≥55 years 35	*p* = 0.5368 26.25 (7.22) 27.41 (8.05)	*p* = 0.7667 31.88 (10.07) 31.06 (11.89)	**0.0087** **0.0358**
BMI (kg/m^2^) Normal (18.5 ≤ 24.99) 34 Overweight (25 ≤ 29.99) 23 Obese (30 ≤ 39.99) 13	***p* = 0.031** 29.26 (6.41) 24.91 (8.32) 23.79 (7.82)	***p* = 0.0009** 36.72 (9.63) 27.82 (8.85) 25.74 (12.40)	**0.0012** 0.2736 0.3109
Menopausal status Premenopausal 26 Postmenopausal 44	*p* = 0.4512 25.89 (7.56) 27.37 (7.69)	*p* = 0.1788 33.87 (10.37) 30.08 (11.18)	**0.0015** 0.0908
Parity status 0 6 1–2 50 3 and more 14	***p* = 0.0385** 28.84 (6.62) 28.12 (7.34) 22.1 (7.67)	*p* = 0.4023 36.79 (7.97) 31.35 (11.24) 29.53 (11.08)	**0.0277** **0.0447** **0.033**
Postmenopausal hormone therapy No 59 Yes 11	*p* = 0.3383 26.44 (7.68) 28.86 (7.28)	*p* = 0.2784 32.01 (10.30) 27.49 (15.22)	**0.0005** 0.5754
Localization of the tumour Right breast 36 Left breast 34	*p* = 0.8924 26.71 (7.31) 26.97 (8.02)	*p* = 0.4601 32.43 (11.19) 30.42 (10.80)	**0.0071** **0.0378**
Diameter of the tumour < 2 cm 48 ≥2 cm < 5 cm 22	*p* = 0.2570 27.58 (7.65) 25.32 (7.49)	*p* = 0.8556 31.63 (11.73) 31.11 (9.50)	**0.0330** **0.0033**
Lymph node status N0 53 N1 17	*p* = 0.2285 26.18 (7.91) 28.77 (6.49)	*p* = 0.4353 30.86 (10.99) 33.34 (11.04)	**0.0057** **0.0494**
TNM staging classification T1 48 T2 22	*p* = 0.2570 27.58 (7.65) 25.32 (7.49)	*p* = 0.8556 31.63 (11.73) 31.11 (9.50)	**0.0330** **0.0033**
Grade according to Elston-Ellis 1 + 2 61 3 9	*p* = 0.7517 26.95 (7.56) 26.03 (8.53)	*p* = 0.5338 31.14 (11.03) 33.74 (10.92)	**0.0056** **0.0180**
Histological type Invasive ductal carcinoma (IDC) 61 Invasive lobular carcinoma (ILC) 9	*p* = 0.1051 26.24 (7.77) 30.67 (5.40)	*p* = 0.0839 30.53 (11.17) 37.33 (7.66)	**0.0047** 0.0506
Molecular type Luminal A (HR+/HER2-/Ki-67 < 20%) 50 Luminal B (HR+/HER2-/Ki-67 ≥ 20%) 16 Luminal B HER2 + (HR+ HER2+) 4	*p* = 0.1418 27.91 (7.60) 24.78 (7.01) 21.66 (8.16)	*p* = 0.5357 30.89 (11.86) 33.91 (8.40) 28.22 (9.57)	0.1234 **0.0018** 0.0679
Staging I 35 II 35	*p* = 0.9218 26.93 (8.09) 26.75 (7.27)	*p* = 0.7694 31.05 (11.99) 31.85 (10.07)	0.098 **0.0015**
Progesterone receptor Negative 5 Positive 65	*p* = 0.8164 27.61 (3.00) 26.77 (7.88)	*p* = 0.7871 30.17 (4.80) 31.56 (11.34)	0.3452 **0.0012**
E-cadherin Negative 5 Positive 65	*p* = 0.0775 32.62 (7.66) 26.37 (7.66)	***p* = 0.0202** 42.3 (6.80) 30.57 (10.8)	**0.0431** **0.0042**
Ki-67 (%) <20 50 ≥20 20	*p* = 0.1076 27.75 (7.40) 24.36 (7.85)	*p* = 0.4026 30.71 (11.30) 33.19 (10.21)	0.0564 **0.0038**

Data are expressed as means ± standard deviation; *p*-values < 0.05 were considered to indicate statistical significance and are marked in bold. BMI: body mass index; N0: no evidence of spread to lymph nodes; N1: spread to axillary lymph nodes; T1: tumour is smaller than 2 cm across; T2: tumour is 2 to 5 cm across; HR+: hormone receptor positive; HER2-: human epidermal growth factor receptor 2 negative; HER2+: human epidermal growth factor receptor 2 positive; Ki67: marker of proliferation Ki-67.

**Table 6:** Results of predictive accuracy for pre-treatment adipokines.

ROC Data	Destimulant	Destimulant
	Pre-Treatment Leptin Concentration ng/mL	Pre-Treatment Adiponectin Concentration ng/mL
AUC	0.599	0.573
Youden index	0.34	0.21
Cut-off point	16.92	28.49
Sensitivity (%)	100.0	77.8
Specificity (%)	34.5	43.1
Positive predictive value (%)	19.1	17.5
Negative predictive value (%)	100.0	92.6
Accuracy (%)	43.3	47.8
*p*-value	0.2497	0.5007

**Table 7:** The median and ROC cut-off point values of the studied parameters before treatment were calculated.

	Pre-Treatment Leptin Concentration ng/mL	Pre-Treatment Adiponectin Concentration ng/mL
Medians	12.38	27.2
ROC cut-off points	16.92	28.49

**Table 8:** Results of predictive accuracy for post-treatment adipokines.

ROC Data	Stimulant	Destimulant
	Post-Treatment Leptin Concentration ng/mL	Post-Treatment Adiponectin Concentration ng/mL
AUC	0.78	0.524
Youden index	0.54	0.29
Cut-off point	26.88	32.88
Sensitivity (%)	88.9	77.8
Specificity (%)	64.9	50.9
Positive predictive value (%)	28.6	20.0
Negative predictive value (%)	97.4	93.5
Accuracy (%)	68.2	54.5
*p*-value	**<0.0001**	0.7886

Significant differences are denoted by bold *p*-values.

**Table 9:** The median and ROC cut-off point values of the studied parameters after treatment were calculated.

	Post-Treatment Leptin Concentration ng/mL	Post-Treatment Adiponectin Concentration ng/mL
Medians	23.66	32.37
ROC cut-off points	26.88	32.88

**Table 10:** Linear regression models for disease-free survival predictors in breast cancer patients.

		Model 1	Model 2	Model 3	Model 4
Pre-Treatment Leptin Concentration	Beta *p*-value	0.0665 0.6010	0.0247 0.8991	−0.2997 0.8782	−0.0793 0.6486
Post-Treatment Leptin Concentration	Beta *p*-value	**−0.3274** **0.0078**	**−0.3895** **0.0031**	**−0.2979** **0.0324**	−0.2413 0.0502
Pre-Treatment Adiponectin Concentration	Beta *p*-value	0.1195 0.3390	0.1743 0.2074	0.2153 0.1211	0.1372 0.2807
Post-Treatment Adiponectin Concentration	Beta *p*-value	0.008 0.9496	0.076 0.5946	0.0109 0.9404	0.0892 0.4901

Model 1 adjusted for age. Model 2 adjusted for age, BMI, parity, menopausal status. Model 3 adjusted for age, BMI, parity, menopausal status and smoking status. Model 4 adjusted for age, BMI, parity, menopausal status, smoking status, tumor stage, tumor diameters, intrinsic type, histological type, nodal involvement. Significant differences are marked with a bold *p*-values.

## Data Availability

The data presented in this study are available in this article.

## Supplementary Materials

The following supporting information can be downloaded at: https://www.mdpi.com/article/10.3390/life12122063/s1, Table S1. Adiponectin concentrations according to the types of surgery and adjuvant therapy in IBrC subjects. Table S2. Adiponectin concentrations regarding types of therapy in IBrC patients.
