# Supplementary material for: High Post-Treatment Leptin Concentration as a Prognostic Biomarker of the High Risk of Luminal Breast Cancer Relapse: A Six-Year Comprehensive Study
Source: Life (Basel). 2022 Dec 8;12(12):2063. doi: 10.3390/life12122063 (PMC9783731; doi:10.3390/life12122063)
Supplement: Supplementary file 1 [file life-12-02063-s001.zip › life-2016020-supplementary.pdf]

Supplementary Materials

Tables S1 and S2 present adiponectin concentrations with regard to the types of therapy. Breast-conserving therapy was linked to higher adiponectin level (*p*=0.0043), comparable effects were obtained with respect to chemotherapy based on anthracykline (*p*=0.0043). Tamoxifen demostrates similar effect on adiponectin level (*p*=0.0076). Application of combine therapy with chemotherapy resulted in an increase of adiponectin concentration (*p*=0.0019).

Table S1. *Adiponectin concentrations according to the types of surgery and adjuvant therapy in IBrC subjects.*

**Feature/** Number of patients	**Pre-Treatment Adiponectin Concentration (ng/mL)**	**Post-Treatment Adiponectin Concentration (ng/mL)**	***P*-value**
**Surgery**	*p*=0.8992	*p*=0.4370	
**BCS + Radiotherapy - BCT**	26.61	33.60	**0.0043**
56	(8.97)	(11.02)	
**Mastectomy**	26.90	30.93	0.2733
14	(7.31)	(10.99)	
**Chemotherapy**	*p*=0.8639	*p*=0.2882	
**Anthracykline**	26.63	33.42	**0.0043**
23	(6.99)	(9.36)	
**Non-anthracykline**	26.23	32.79	0.2733
4	(9.47)	(15.45)	
**No**	26.99	30.32	<u>0.0563</u>
43	(7.91)	(11.41)	

**Endocrine therapy**	*p*=0.5380	*p*=0.6086	
**Tamoxifen**	26.74	31.73	**0.0076**
40	(8.06)	(12.10)	
**Inhibitor aromatase**	28.36	30.36	0.4074
17	(7.51)	(10.17)	
**Tamoxifen and inhibitor**	24.03	26.17	0.4990
**aromatase**	(6.39)	(5.67)	
7			
**Other type**	24.61	38.77	0.1797
4	(0.88)	(8.33)	
**No**	27.73	39.73	0.1797
2	(10.04)	(8.40)	

Data are expressed as means ± standard deviation; p-values <0.05 were considered to indicate statistical significance and are marked in bold. BCS: breast-conserving surgery; BCT: breast-conserving therapy.

Table S2. *Adiponectin concentrations regarding types of therapy in IBrC patients.*

**Feature/** Number of patients	**Pre-Treatment Adiponectin Concentration (ng/mL)**	**Post-Treatment Adiponectin Concentration (ng/mL)**	***P*-value**
	p=0.8451	*p*=0.2193	
**Monotherapy**	27.73	39.73	0.1797
2	(10.04)	(8.40)	
**Combination therapies with**	26.57	33.32	**0.0019**
**chemotherapy**	(7.22)	(10.14)	
26			
**Combination therapies with**	26.95	29.84	0.1156
**hormone therapy**	(7.94)	(11.42)	
42			

Data are expressed as means ± standard deviation; p-values <0.05 were considered to indicate statistical significance and are marked in bold.
